# Complement regulatory proteins: Candidate biomarkers in differentiating tuberculosis pleural effusion

**DOI:** 10.3389/fimmu.2023.1073884

**Published:** 2023-02-03

**Authors:** Huan Tang, Xinyue Hu, Li Li, Shuanglinzi Deng, Yuanyuan Jiang, Lisha Luo, Runjin Cai, Yifei Yang, Chendong Wu, Xiaoxiao Gong, Juntao Feng

**Affiliations:** ^1^ Department of Respiratory Medicine, Key Cite of National Clinical Research Center for Respiratory Disease, Xiangya Hospital, Central South University, Changsha, Hunan, China; ^2^ National Clinical Research Center for Geriatric Disorders, Xiangya Hospital, Central South University, Changsha, Hunan, China; ^3^ Division of Environmental and Occupational Health Sciences, Department of Medicine, National Jewish Health, Denver, CO, United States

**Keywords:** complement, sCD46, sCD55, sCD59, tuberculosis pleural effusion

## Abstract

**Background and aims:**

Complement activation is essential for tuberculosis pleural effusion. However, little is known about the value of complement regulatory protein (CD46, CD55, and CD59) in the differential diagnosis of tuberculosis.

**Materials and methods:**

Ninety-nine patients with exudative pleural effusion admitted to Xiangya Hospital of Central South University from June 1, 2021to November 14, 2022 were enrolled. The expression levels of soluble CD46 (sCD46), soluble CD55 (sCD55), and soluble CD59 (sCD59) in pleural effusion were quantified by enzyme-linked immunosorbent assay, and the receiver operating characteristic (ROC) curves were plotted to evaluate the diagnostic and co-diagnostic values.

**Results:**

The ADA level is higher in TPE patients than non-TPE patients. It is well-found that TPE patients had lower levels of sCD46, sCD55, and sCD59 compared with non-TPE patients. Moreover, the expression of sCD46, sCD55, and sCD59 in pleural effusion was negatively correlated with ADA. In addition, the diagnostic efficacy of sCD46, sCD55 and sCD59 was comparable to that of ADA, with 0.896, 0.857, 0.858 and 0.893, respectively. Furthermore, combine detection of sCD46, sCD55, sCD59 and ADA could improve the diagnostic accuracy.

**Conclusions:**

Complement regulatory factors (CD46, CD55, and CD59) were validated by this project to be promising candidate biomarkers for the diagnosis of TPE with high accuracy. The combination of the CD46, CD55, and CD59 and ADA assay exist a better diagnostic value in TPE.

## Introduction

Tuberculosis (TB), an infectious disease caused by Mycobacterium tuberculosis (M*tb*), is the leading cause of death from a single infectious disease and a major global health problem (1). Depending on the site of infection, TB is typically divided into intrapulmonary and extrapulmonary TB. Tuberculosis pleural effusion (TPE), the second most common form of extrapulmonary TB, is the main cause of exudative pleural effusion (2, 3), which is very common in clinical practice. Additionally, the differentiation of TPE from other types of exudative pleural effusion, such as malignant pleural effusion (MPE), and parapneumonic pleural effusion (PPE) is also a big challenge (4).

The diagnosis of TPE currently relies on positive Ziehl-Neelsen staining, or M*tb* culture of pleural effusion, or a granuloma in pleural biopsy specimens. However, there are some limitations in clinical practice, as the rate of positive Ziehl-Neelsen staining and M*tb* culture of PE is extremely low and requires long-term culture in the specialized laboratory. In addition, pleural biopsy is an invasive procedure with risks of complications (5, 6). Of note, in clinical practice, adenosine deaminase (ADA) is a common biomarker for TPE diagnosis, but there are still some TPE patients with low ADA levels (6, 7). Moreover, some serum or pleural effusion biomarkers such as lactate dehydrogenase (LDH), erythrocyte sedimentation rate (ESR), and Interferon-gamma (IFN-γ) can also be used as auxiliary guide diagnosis in TPE but with limited sensitivity and specificity. Therefore, it is necessary to explore new and novel non-invasive biomarkers with high sensitivity and specificity.

In recent years, several studies have identified the involvement of the complement system in the occurrence and development of tuberculosis, and some complement components such as C1q have been highlighted as candidate diagnostic biomarkers of tuberculosis and TPE, as well as a serum biomarker for the detection of active tuberculosis (8–10). Of note, the complement regulatory proteins CD55, CD46 and CD59 can regulate the complement system and prevent its excessive activation. A previous study has found lower levels of sCD55 and sCD97 TPE patients than in MPE patients (11). Other studies have identified CD46 as a T cell costimulatory molecule for T cell activation and inducing Type 1 regulatory (Tr1) cells and CD46-dependent negative regulatory mechanisms impaired T cell responses which is essential for immune defense against mycobacteria (12). However, information on whether CD46, CD55, and CD59 can serve as candidate biomarkers for TPE is rather limited. Thus, we conducted this study to determine the exact role of complement regulatory proteins CD46, CD55, and CD59 in the diagnosis of TPE.

## Methods

### Study population

**Figure 1:**
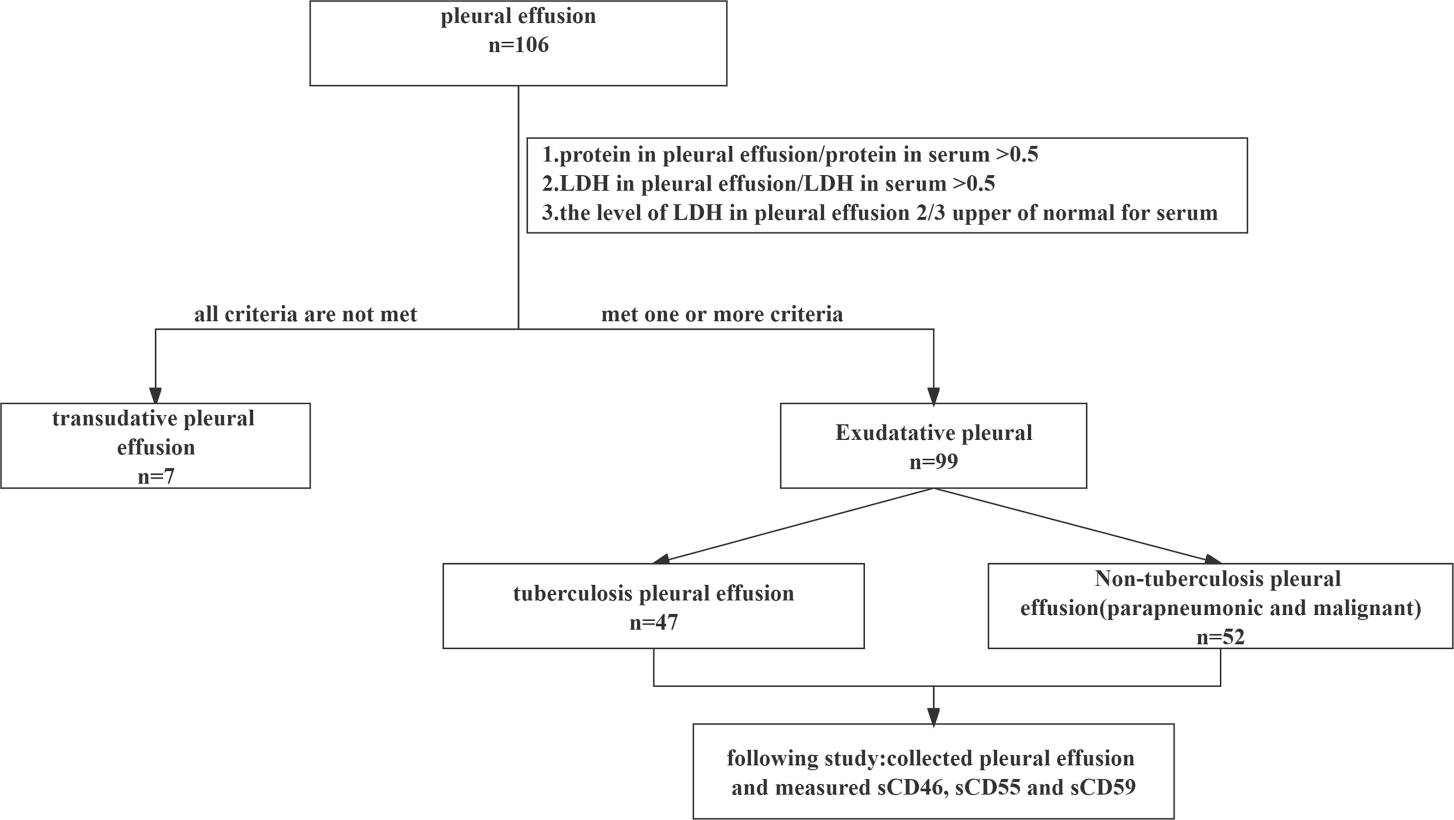
Flow chart showing the selection of patients.

Ninety-nine patients with pleural effusion who met the inclusion criteria from June 2021 to November 2022 in Xiangya Hospital of Central South University, Changsha, Hunan, were included in the study. According to the *Light* criteria, all patients were diagnosed with exudative pleural effusion (13). There were 47 cases in the TPE group and 52 cases in the non-TPE group (12 cases with PPE and 40 cases with MPE) (**Figure 1**). The diagnostic criteria for tuberculosis pleural patients were listed as follows: positive Ziehl-Neelsen staining or *Mtb* culture from pleural effusion or the presence of granuloma in the pleural biopsy specimens, non-tuberculosis patients include MPE and PPE. Of these, MPE was diagnosed in patients with malignant cells found in pleural effusion or/and pleural biopsy specimens, while PPE was diagnosed in those found in pleural effusion caused by bacterial pneumonia, lung abscess, and bronchiectasis. Other information including demographics (gender, age, different complication) and laboratory results (ADA, C-reactive protein, LDH, ESR and T-SPOT) were collected from electronic medical records.

### Pleural effusion collection

Pleural effusions were collected and centrifuged at 1500 rpm for 10 minutes. The supernatant was transferred into a new tube and stored at -80 °C refrigerator for later sCD55, sCD46, and sCD59 measurements.

### Measurement of sCD55, sCD46, and sCD59

The concentration of the sCD55, sCD46, and sCD59 in pleural effusion was measured by enzyme-linked immunosorbent assay (ELISA) kits (*RENJIEBIO, Shanghai, China*) according to the manufacturer’s introductions.

### Statistical analysis

The collected data were divided into categorical variables and continuous variables. After the examination of the Kolmogorov-Smirnov test, Continuous variables following normal distribution are described as the mean (standard deviation, SD) and Continuous variables with skewed distribution are described as the median (interquartile range, IOR). Categorical variables are described as the frequency (%). Differences in continuous statistics between groups were compared using the students t-test or Mann-Whitney U-test, while the Pearson’s chi-squared test was used for comparing categorical data. ROC analysis and consistency analysis was used to evaluate the diagnostic value of sCD46, sCD55, and sCD59. Positive likelihood ratio (PLR), negative likelihood ratio (NLR), positive predictive value (PPV), and negative predictive value (NPV) were calculated by MedCalc software. Consistency analysis was employed to analyze the diagnostic consistency of ADA with sCD46, sCD55 and sCD59. *P* < 0.05 was considered as statistically significant.

## Results

### Clinical and demographic characteristics information

**Figure 2:**
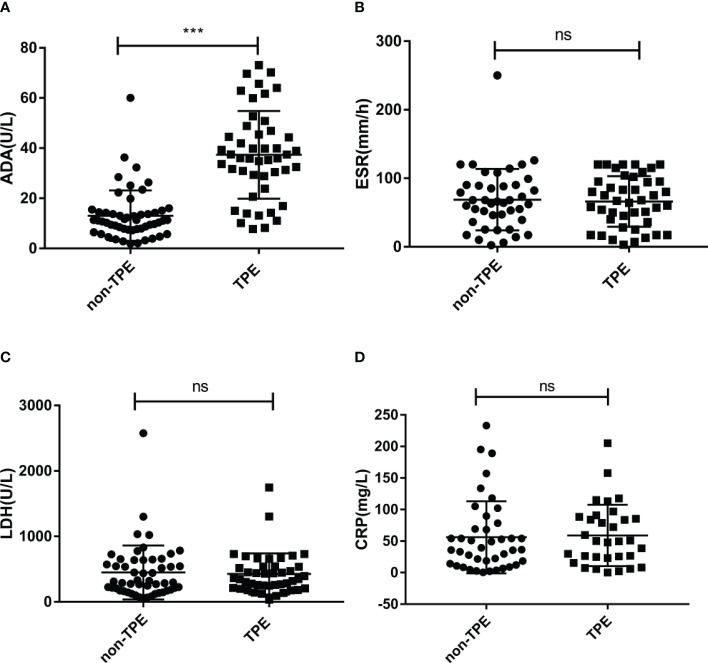
Concentration of ADA, LDH, ESR and CRP in TPE (N=47) and non-TPE(N=52) patients. Comparison of ADA **(A)**, ESR **(B)**, LDH **(C)**, CRP **(D)** level in TPE and those in non-TPE. ^∗∗∗^ *P* < 0.001, ns *P* > 0.05.

Baseline demographic information and laboratory data of TPE and non-TPE patients are shown in **Table 1**. Of these patients, 47 with TB infection (TPE group), and 52 with other diseases such as MPE and PPE (non-TPE group) were included. The average age between TPE and non-TPE groups was 53.8± 2.6 vs 58.0 ± 2.0. The ratio of females/males between these two groups was 15/47 (31.9%) vs 15/52 (28.9%). The differences in age and gender between the two groups were not statistically significant. Compared to non-TPE patients, TPE patients had an obvious higher ADA level (37.36 ± 2.55 U/L vs 13.05 ± 1.39 U/L). Besides, the positive rate of T-SPOT was also significantly higher in the TPE group than in the non-TPE group (87.2% vs 28.9% *p*<0.0001). However, other baseline information and biochemical makers including different complication, LDH, ESR and C-reactive protein (CRP) levels exist no significant difference between patients with or without TPE (**Table 1** and **Figures 2A–D**).

**Table 1:** Baseline demographic information and laboratory test.

Variables	non-TPE (n=52)	TPE (n=47)	P value
Gender (female/male)	15/52 (28.9%)	15/47 (31.9%)	0.740
Age, years	58.0 ± 2.0	53.8± 2.6	0.217
Complication			
COPD	3/52 (5.7%)	2/47 (4.3%)	0.530
Diabetes	7/52 ( (13.5%)	5/47 (10.6%)	0.589
Hepatitis	1/52 (1.9%)	2/47 (4.3%)	0.610
Kidney disease	8/52 (15.4%)	4/47 (8.5%)	0.359
Bronchiectasis	1/52 (1.9%)	2/47 (4.3%)	0.610
Protein (g/L)	42.44 ± 1.93	58.93 ± 8.59	0.823
LDH (U/L)	448.6 ± 57.31	425.7 ± 47.94	0.766
ADA (U/L)	13.05 ± 1.39	37.36 ± 2.55	< 0.0001
CRP (mg/L)	56.09 ± 8.92	58.93 ± 8.59	0.823
ESR (mm/h)	68.68 ± 6.92	66.14 ± 5.63	0.776
T-SPOT-positive	15/52 (28.9%)	41/47 (87.2%)	< 0.0001

COPD, chronic obstructive pulmonary disease; LDH, lactate dehydrogenase; ADA, adenosine deaminase; CRP, C-reactive protein; ESR, erythrocyte sedimentation rate; T-SPOT, interferon gamma release assay. Data are shown as n/N (%) or median (interquartile range mean ± standard deviation unless specified otherwise).

### TPE patients display lower concentration of sCD46, sCD55, and sCD59

**Figure 3:**
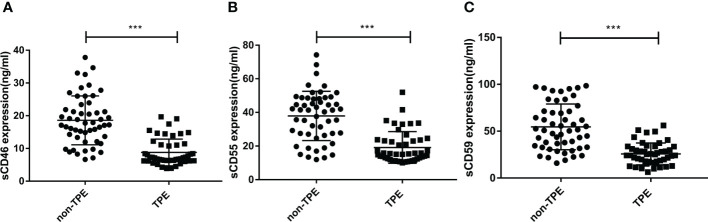
Concentration of sCD46, sCD55 and sCD59 in TPE (N=47) and non-TPE(N=52) patients. The level of sCD46, sCD55 and sCD59 in pleural effusion were detected by ELISA. Comparison ofsCD46 **(A)**, sCD55 **(B)**, sCD59 **(C)** level in TPE and those in non-TPE. ^∗∗∗^ *P* < 0.001.

To further evaluate the significance of complement regulatory proteins in TPE, sCD46, sCD55, and sCD59 levels were detected in supernatant from pleural effusion by ELISA. As shown in **Table 2** and **Figures 3A–C**, without considering other factors, the sCD46, sCD55 and sCD59 concentrations were lower in TPE patients compared to non-TPE patients. In details, sCD46, sCD55, and sCD59 levels in the non-TPE patients were nearly 2 times higher than those in TPE patients.

**Table 2:** The concentration of sCD46, sCD55, and sCD59 in pleural effusion.

Variables	non-TPE(n=52)	TPE(n=47)	P value
sCD46(ng/ml)	18.58 ± 1.04	8.77 ± 0.60	< 0.0001
sCD55(ng/ml)	37.89 ± 2.04	19.12 ± 1.38	< 0.0001
sCD59(ng/ml)	54.58 ± 3.38	25.73 ± 1.71	< 0.0001

Data are shown as median (interquartile range mean ± standard deviation).

### Negatively correlation exist between sCD46, sCD55, and sCD59 with ADA

**Figure 4:**
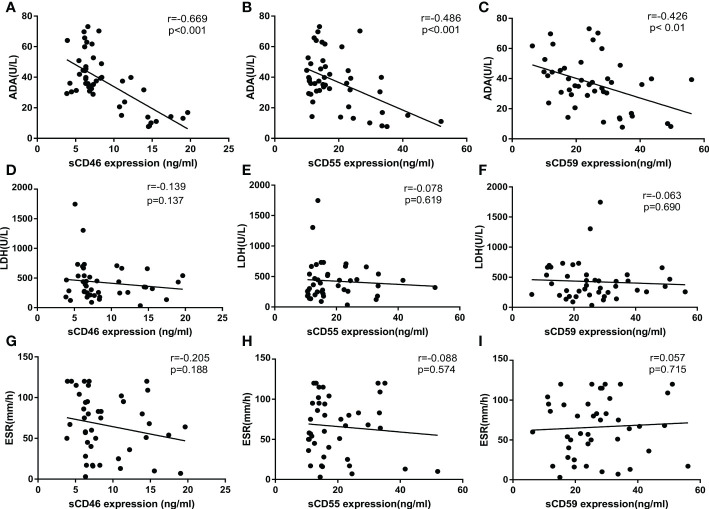
Correlation analysis of sCD55, sCD46, sCD59, ADA, LDH, ESR. **(A–C)** Correlation analysis of sCD46, sCD55 and sCD59 with ADA(N=99). **(D–F)** Correlation analysis of sCD46, sCD55 and sCD59 with LDH(N=99). **(G–I)** Correlation analysis of sCD46, sCD55 and sCD59 with ESR(N=99). Correlations were determined by Pearson correlation coefficients. sCD46 sCD55 and sCD59 content in PE were negatively correlated with ADA (r = -0.668, *P< 0.001*, r = -0.486, *P< 0.001* and r = -0.426, *P < 0. 01* respectively), which indicated that sCD46 sCD55 and sCD59 could turn into an effective biomarker for PE differential diagnosis.

It is well known that ADA, LDH, and ESR are commonly used as biochemical indicators to distinguish TPE from non-TPE, especially ADA around the most significant. To explore the correlation between sCD46, sCD55, sCD59, and ADA, ESR, and LDH separately, biochemical information was collected from the medical record, then correlation analysis was used to investigate the correlation among sCD46, sCD55, sCD59, and ADA, ESR, LDH. Notably, we found that sCD46, sCD55, and sCD59 were inversely correlated with ADA (**Figures 4A–C**), but no correlations were found between sCD46, sCD55, sCD59, and ESR, LDH (**Figures 4D–I**).

### Diagnostic values of sCD46, sCD55, and sCD59 is similar with ADA in TPE

**Figure 5:**
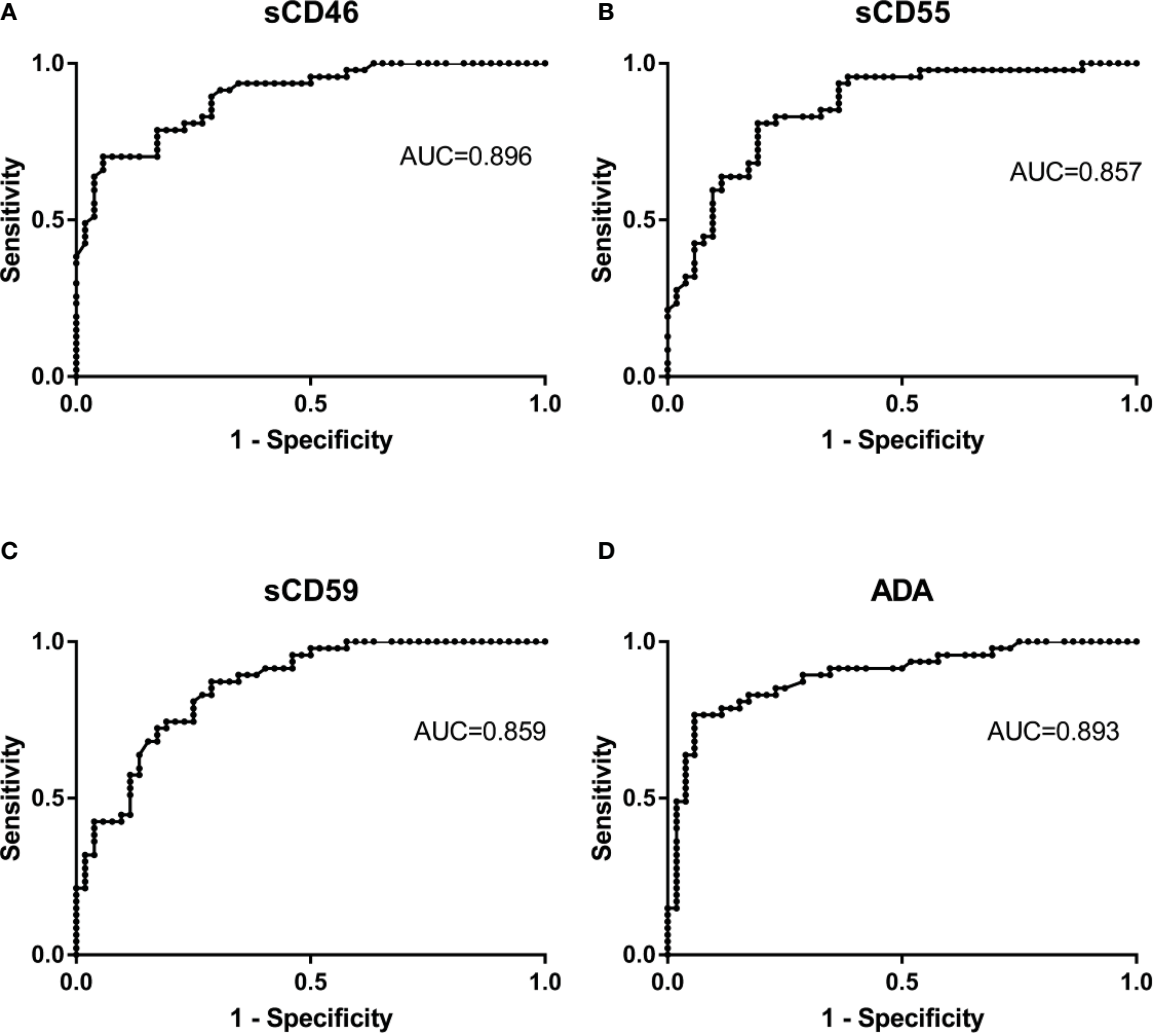
Receiver operating characteristic curves of Pleural Fluid sCD46, sCD55, and sCD59 and ADA for TPE. The ROC curves show the diagnostic value of sCD46 **(A)**, sCD55 **(B)**, sCD59 **(C)** and ADA **(D)** for TPE.

To clarify the diagnostic value of sCD46, sCD55, and sCD59 for PE, this study plotted subject ROC curve to assess the sensitivity, specificity, PPV, NPV, PLR, and NLR of these biomarkers. In the general patients, with a cut of 8.41 U/L, the AUC, sensibility, specificity, PLR, NLR, PPV, and NPV of sCD46 to discriminate TPE and non-TPE cases were 0.896, 70.21, 94.23, 12.17, 0.32, 91.7, and 77.8 respectively (**Figure 5A** and **Table 3**). The AUC sensibility, specificity, PLR, NLR, PPV, and NPV of sCD55 is 0.857, 80.85, 80.77, 4.2, 0.24, 79.2, and 82.4 respectively (**Figure 5B** and **Table 3**). Moreover, the AUC sensibility, specificity, PLR, NLR, PPV, and NPV of sCD59 is 0.858, 87.23, 71.15, 3.02, 0.18, 73.2, and 86.0 respectively (**Figure 5C** and **Table 3**). Meanwhile, the AUC of ADA was 0.893 (**Figure 5D**), and there was no significant difference of the AUC between ADA and sCD46 (*z*=0.07, *p=0.94*), sCD55 (*z*=0.82, *p=0.41*), sCD59 (*z*=0.74, *p=0.46*). In addition, we found a good consistence between ADA and sCD46 (*kappa=0.787, p<0.001*), and a moderate consistence between ADA and sCD55 (*kappa=0.553, p<0.001*). While the consistence between ADA and sCD59 was poor (*kappa=0.339, p<0.001*).

**Table 3:** Diagnostic performance of sCD46, sCD55 and sCD59 in PF in differentiating between patients with TPE and those with non-TPE.

Variable	Cut-off value	AUC (95%CI)	Sensitivity (95%CI)	Specificity (95%CI)	PLR (95%CI)	NLR (95%CI)	PPV (95%CI)	NPV (95%CI)
ADA	28.4	0.893 (0.815-0.946)	76.60 (62.0-87.7)	94.23 (84.1-98.8)	13.28 (4.38-40.28)	0.25 (0.15-0.42)	92.3 (79.8-97.3)	81.7 (72.6-88.2)
sCD46	8.41	0.896 (0.818-0.948)	70.21 (55.1-82.7)	94.23 (84.1-98.8)	12.17 (3.99-37.08)	0.32 (0.20-0.49)	91.7 (78.3-97.1)	77.8 (69.2-84.5)
sCD55	24.56	0.857 (0.773 -0.919)	80.85 (66.7-90.9)	80.77 (67.5-90.4)	4.2 (2.37-7.47)	0.24 (0.13-0.43)	79.2 (68.2-87.1)	82.4 (71.9-89.5)
sCD59	37.55	0.858 (0.774 -0.921)	87.23 (74.3-95.2)	71.15 (56.9-82.9)	3.02 (1.95-4.70)	0.18 (0.08-0.39)	73.2 (63.8-80.9)	86.0 (74.1-93.0)

AUC, area under the curve; PLR, positive likelihood ratio; NLR, negative likelihood ratio; PPV, positive predictive value; NPV, negative predictive value.

### Combined detection of sCD46, sCD55, sCD59, and ADA improve diagnostic values for PE differential diagnosis

**Figure 6:**
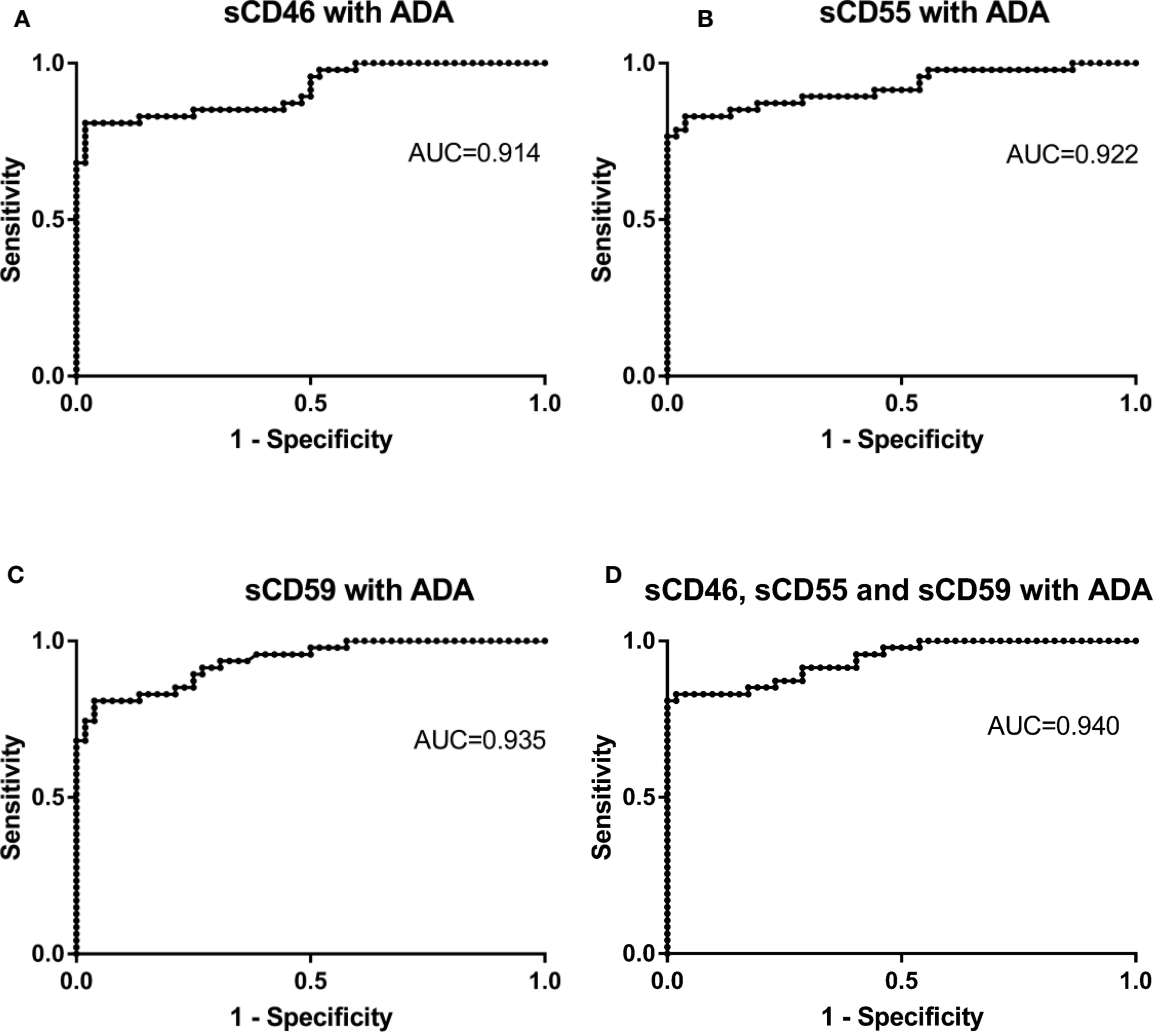
Receiver operating characteristic curves of combine detection of sCD46. sCD55, sCD59 and ADA for differential diagnosis in TPE (N=47) and non-TPE(N=52). **(A)** the ROC curves of combined detection of Pleural Fluid sCD46 and ADA. **(B)** the ROC curves of combined detection of Pleural Fluid sCD55 and ADA. **(C)** the ROC curves of combined detection of Pleural Fluid sCD59 and ADA. **(D)** the ROC curves of combined detection of Pleural Fluid sCD46, sCD55, sCD59, and ADA.

To further improve the diagnosis accuracy, we validated whether the combined detection of sCD46, sCD55, sCD59 and ADA could better identify patients with TPE and Non-TPE patients. The current results suggest that the used of ADA in combination with sCD46, sCD55, or sCD59, as well as the combined detection of these four biomarkers, improves diagnostic accuracy with the AUCs of 0.914, 0.922, 0.935, and 0.940, respectively (**Figures 6A–D**). Furthermore, the AUC of sCD59 with ADA and combine detection of these four biomarkers were higher than that of ADA alone (*z*=2.204, *p*=0.041, *z*=2.194, *p*=0.028). But there was no significant difference of the AUC between sCD46 with ADA, sCD55 with ADA and ADA alone (*z*= 0.918, *p*=0.359, *z*=1.292, *p*=0.196). In addition, PLR of combined detection of complement regulatory proteins with ADA were 42.04, 21.57, 21.02 and 43.15. The PPV (97.4%, 95.1%, 95.0%, 97.5%)and NPV (85.0%, 86.2%, 84.7% and 86.4%) were found in our study (**Table 4**).

**Table 4:** Diagnostic performance of combined detection of sCD46, sCD55 and sCD59 with ADA in PF.

Variable	Sensitivity (95%CI)	Specificity (95%CI)	PLR (95%CI)	NLR (95%CI)	PPV (95%CI)	NPV (95%CI)
sCD46 with ADA	80.85 (66.7-90.9)	98.08 (89.7-100)	42.04 (6.01-294.34)	0.20 (0.11-0.35)	97.4 (84.4-99.6)	85.0 (75.9-91.1)
sCD55 with ADA	82.98 (69.2-92.4)	96.15 (86.8-99.5)	21.57 (5.51-84.49)	0.18 (0.094-0.33)	95.1 (83.3-98.7)	86.2 (76.8-92.2)
sCD59 with ADA	80.85 (66.7-90.9)	96.15 (86.8-99.5)	21.02 (5.36-82.41)	0.20 (0.11-0.36)	95.0 (82.9-98.7)	84.7 (75.5-90.9)
sCD46, sCD55 and sCD59 with ADA	82.98 (69.2-92.4)	98.08 (89.7-100.0)	43.15 (6.17-301.88)	0.17 (0.092-0.33)	97.5 (84.8-99.6)	86.4 (77.2-92.3)

PLR, positive likelihood ratio; NLR, negative likelihood ratio; PPV, positive predictive value; NPV, negative predictive value.

## Discussion

There were about an estimated 10.6 million people ill with tuberculosis and 1.6 million people died in 2021 in 2021 according to WHO’s global tuberculosis report 2022 (14). TPE is the second most common form of extrapulmonary-TB but with low sensitivity of detection of M*tb* in PE (2). Currently, the gold standard of TPE diagnosis relies on M*tb* culture of PF or pleural biopsy specimens but with various limitations such as low positive-culture rate of invasive procedures culture and complications (5). Additionally, it is well-founded that ADA is a common clinical biomarker to diagnose TPE, but in some TPE patients, there is no significant elevation of ADA levels in pleural fluid (PF) (6, 15). The purpose of this project was to propose and evaluate several new and novel biomarkers for differentiate TPE diagnosis.

In recent years, complement activation has played an important role in various diseases. It consists of more than 50 kinds of protein that either circulate in the fluid phase or bind to the cell membrane. The complement system activated by M*tb* infection can also be described as a bridge between innate immunity and adaptive immunity (16, 17). Complement system activation leads to C1q, C3a, and C5a cleavage and the formation of membrane attack complex (MAC) (18). Our previous study found complement system activity in TPE patients with higher levels of complement components C3a, C5a, C1q, and MAC than in transudative pleural effusion. Moreover, C3a and C5a are involved in M*tb* infection by inducing inflammatory cytokine secretion, orchestrating Th17 response, and stimulating PMC to generate CCL2, CCL7, and CX3CL1 which in turn recruit CD14^+^CD16^+^ monocyte (19, 20). In addition, several studies have also found that complement component C1q in pleural effusion can be used as a candidate biomarker for the diagnosis of TPE and serum C1q can also be used as an indicator for active tuberculosis infection (8–10). Therefore, it is well-speculated complement system related factors play an important role in TPE.

Complement regulatory proteins, including CD46, CD55 and CD59, play a significant role in preventing the complement system from over-activation by decreasing the production of C3a, C5a, and MAC. CD46, CD55 and CD59, as phosphatidylinositol-linked membrane proteins, are widely expressed on various cell membranes, including blood, mesenchymal, epithelial and endothelial cells (21). The previous study has found higher CD46, CD55, and CD59 levels in monocytes of COVID-19 patients than in healthy people, especially CD55 levels correlated with plasma inflammatory markers such as CRP and serum amyloid A during acute infection (22). Moreover, the expression of CD46, CD55, and CD59 in blood cells also increased after injury (23). Another study presented that CD55 and its ligand CD97 can be used to discriminate between TPE and MPE (11). Besides, CD46 is sought to be a T cell costimulatory molecule for T cell activation and inducing Type 1 regulatory cells (Tr1) in tuberculosis infection (12). However, little is known about whether sCD46, sCD55, and sCD59 can be used for differentiating TPE and non-TPE cases.

Similar to previous study (11), we detected lower sCD55 levels in TPE compared with non-TPE cases. Interestingly, we also found lower sCD46 and CD59 expression in TPE cases than in non-TPE patients. The lower levels of sCD46, sCD55, and sCD59 may be due to excessive complement regulator consumption and indicate the complement system over-activation and predict immune system activation (24). The levels of soluble complement regulatory proteins were related with some several chronic inflammatory diseases including SLE and RA, and related with disease activity (25–27). Whether the lower level of sCD46, sCD55 and sCD59 in TPE patients related with disease process needed to further researched. Then, we found the levels of sCD46, sCD55, and sCD59 were negatively correlated with the level of ADA, a most common biomarker for TPE diagnosis. CD46, CD55, and CD59 are great diagnostic candidate biomarkers for discriminating TPE diagnosis, and further subgroup analysis was processed according to diagnostic values and combination detection analysis. While no correlation were found between sCD46, sCD55, sCD59 and ESR, LDH in our study, which may cay due to the limited sensibility and specificity of ESR and LDH in diagnosis of TPE.

There were no significant differences in AUC between these three biomarkers and ADA, suggesting that sCD46, sCD55, and sCD59 were as effective as ADA. Furthermore, a kappa value of 0.787 showed a good consistency between sCD46 and ADA, and further expand the sample size may improve the consistency between sCD55, sCD59 and ADA. Our data also showed that ADA and sCD46 have a good diagnostic specificity 94.23%, PLR (13.28 and 12.17), indicated that the probability of positive ADA and sCD46 in TPE patients were 13.28 and 12.17-fold higher than non-TPE patients. Moreover, ADA and sCD46 have high PPV (92.3% and 91.7%), further indicated the false-negative rates were low.

While these biomarkers have limited sensibility, NLR and NPV. To improve the diagnostic accuracy, we combined detection of sCD46, sCD55, sCD59, and ADA with a higher AUC compared to ADA alone. we found that combined detection of complement regulatory proteins with ADA have a better sensibility and specificity. Furthermore, high PLR (42.04, 21.57, 21.02 and 43.15) of combined detection of complement regulatory proteins with ADA found in this study suggesting that these biomarkers are sufficiently high enough for diagnosis. In addition, higher PPV(97.4%, 95.1%, 95.0% and 97.5%) and NPV(85%, 86.2%, 84.7% and 86.4%) of combined detection further indicate that lower false-positive rates and false-negative rates. Our data also showed that combined detection of sCD59 with ADA and combined detection of sCD46, sCD55 and sCD59 with ADA have better diagnostic value than ADA, while there was no significant difference between combined detection of sCD46 and sCD55 with ADA and ADA detection alone, which mainly due to the small sample size.

There is some limitation in the current study. Firstly, our sample size is limited, especially the lack of PF patients with autoimmune diseases. Moreover, Patients included in this study were older and lacked younger patients, which may affect the level of ADA (28, 29).

In summary, our present data suggested that TPE patients have a lower level of PF sCD46, sCD55, and sCD59 compared with non-TPE patients. Both the sensitivity and specificity of pleural effusion sCD46, sCD55, sCD59, and ADA perform with equivalent diagnostic effects, and combine detection improved the diagnostic accuracy, suggesting that they can be used as biomarkers for differentiating TPE and non-TPE.

## Data availability statement

The raw data supporting the conclusions of this article will be made available by the authors, without undue reservation.

## Ethics statement

The studies involving human participants were reviewed and approved by the Medical Ethics Committee of the Xiangya Hospital of Central South University. The patients/participants provided their written informed consent to participate in this study. Written informed consent was obtained from the individual(s) for the publication of any potentially identifiable images or data included in this article.

## Author contributions

HT and XH performed the experimental work, collected and analyzed the data, and wrote the manuscript. SD, LL, YJ, LSL, RC, YY, CW, and XG helped collected and analyzed the data. JF designed the study, supervised the study and critically revised the manuscript. HT and XH equally contributed to the paper. All authors contributed to the article and approved the submitted version.
